# Supplementary material for: Profiling Risk Factors for Household and Community Spatiotemporal Clusters of Q Fever Notifications in Queensland between 2002 and 2017
Source: Pathogens. 2022 Jul 25;11(8):830. doi: 10.3390/pathogens11080830 (PMC9332293; doi:10.3390/pathogens11080830)
Supplement: Supplementary file 1 [file pathogens-11-00830-s001.zip › pathogens-1781923-supplementary.pdf]

Supplementary Materials

Table S1. Q fever clustering using space-time analysis in Queensland between 2002 and 2017

ID	Radius (Km)	LLR	P value	Observed cases	Expected cases	RR	Population	LGA	YEAR	Cluster
1	0.000	51.46	0.00	6	0.00	14453.22	86	Paroo Shire	2002	H
2	0.000	40.48	0.00	5	0.00	8920.27	118	Gympie Regional	2008	H
3	1.350	40.16	0.00	6	0.00	2196.07	566	Murweh Shire	2015	C
4	0.785	36.87	0.00	5	0.00	4334.63	235	Maranoa Regional	2006	C
5	9.664	33.77	0.00	8	0.04	184.39	9146	Townsville City	2012	C
6	0.784	31.57	0.00	5	0.00	1501.31	713	Balonne Shire	2002	H
7	0.000	25.45	0.00	3	0.00	13131.61	93	Ipswich City	2013	H
8	0.000	25.28	0.00	2	0.00	840911.35	1	South Burnett Regional	2013	H
9	0.000	24.88	0.00	3	0.00	10878.86	116	Gold Coast City	2003	H
10	0.000	24.59	0.00	3	0.00	9871.82	65	Toowoomba Regional	2014	H
11	0.000	24.18	0.00	3	0.00	8600.28	142	Gympie Regional	2002	H
12	0.000	23.86	0.00	2	0.00	413562.96	1	Ipswich City	2010	H
13	0.000	23.58	0.00	3	0.00	7052.61	88	South Burnett Regional	2017	H
14	0.362	23.15	0.00	3	0.00	6106.20	100	Murweh Shire	2017	C
15	7.081	22.91	0.00	4	0.00	835.07	1025	Barcaldine Regional	2015	C
16	0.000	22.58	0.00	3	0.00	5045.77	123	Southern Downs Regional	2015	H
17	0.000	19.67	0.01	2	0.00	50861.57	16	Toowoomba Regional	2015	H
18	0.000	18.70	0.02	2	0.00	31299.43	26	Southern Downs Regional	2009	H
19	0.000	18.11	0.02	2	0.00	23251.00	35	Southern Downs Regional	2003	H
20	6.123	17.30	0.05	3	0.00	868.01	715	Gympie Regional	2002	C
21	0.000	17.03	0.06	2	0.00	13563.09	60	Maranoa Regional	2004	H
22	0.000	16.96	0.07	2	0.00	13125.57	62	Brisbane City	2004	H
23	0.000	16.79	0.08	2	0.00	12013.02	70	Noosa Shire	2002	H
24	0.000	16.68	0.09	2	0.00	11363.67	74	Western Downs Regional	2009	H
25	0.000	16.60	0.10	2	0.00	10920.93	77	Western Downs Regional	2013	H
26	0.000	16.57	0.10	2	0.00	10780.91	78	Bundaberg Regional	2012	H
27	0.000	16.39	0.13	2	0.00	9846.74	42	Bundaberg Regional	2004	H
28	0.000	16.29	0.15	2	0.00	9353.85	87	Gold Coast City	2016	H
29	0.609	16.28	0.15	3	0.00	617.54	1005	Maranoa Regional	2002	C
30	0.000	16.15	0.18	2	0.00	8726.16	49	Blackall Tambo Regional	2002	H
31	0.000	16.07	0.20	2	0.00	8389.54	97	Western Downs Regional	2012	H
32	0.000	15.81	0.26	2	0.00	7376.41	114	Townsville City	2017	H
33	0.000	15.78	0.27	2	0.00	7255.49	57	Western Downs Regional	2011	H
34	0.000	15.71	0.29	2	0.00	7015.39	116	Scenic Rim Regional	2016	H

ID	Radius (Km)	LLR	P value	Observed cases	Expected cases	RR	Population	LGA	YEAR	Cluster
35	0.000	15.61	0.32	2	0.00	6670.37	62	Ipswich City	2015	H
36	2.487	15.47	0.36	2	0.00	6212.10	131	Bundaberg Regional	2014	C
37	0.000	15.28	0.43	2	0.00	5665.25	73	Blackall Tambo Regional	2002	H
38	0.000	15.24	0.45	2	0.00	5535.95	147	Gold Coast City	2013	H
39	0.855	15.19	0.46	3	0.01	429.50	1445	Ipswich City	2002	C
40	0.000	15.13	0.49	2	0.00	5234.97	79	Fraser Coast Regional	2016	H
41	4.702	14.98	0.52	2	0.00	4860.76	173	Southern Downs Regional	2013	C
42	0.116	14.96	0.53	2	0.00	4808.87	86	Southern Downs Regional	2013	C
43	0.000	14.95	0.53	2	0.00	4786.97	170	Ipswich City	2006	H
44	0.000	14.82	0.59	2	0.00	4496.85	187	Mackay Regional	2017	H
45	1.662	14.82	0.59	2	0.00	4495.25	92	Toowoomba Regional	2015	C
46	0.000	14.76	0.61	2	0.00	4351.79	187	Central Highlands Regional	2004	H
47	0.000	14.72	0.63	2	0.00	4263.53	97	Lockyer Valley Regional	2011	H
48	0.000	14.68	0.64	2	0.00	4194.77	97	Bundaberg Regional	2002	H
49	0.000	14.60	0.67	2	0.00	4015.17	103	South Burnett Regional	2010	H
50	0.355	14.51	0.70	2	0.00	3852.09	111	Balonne Shire	2013	C
51	0.000	14.41	0.74	2	0.00	3659.85	113	Tablelands Regional	2017	H
52	0.849	14.41	0.74	2	0.00	3659.85	113	Charters Towers Regional	2016	C
53	0.000	14.36	0.75	2	0.00	3565.20	116	Sunshine Coast Regional	2002	H
54	0.000	14.33	0.76	2	0.00	3507.69	116	South Burnett Regional	2017	H
55	0.000	14.15	0.82	2	0.00	3205.91	129	Gympie Regional	2008	H
56	0.000	13.98	0.87	2	0.00	2954.02	140	Logan City	2006	H
57	0.136	13.91	0.89	2	0.00	2852.16	145	Rockhampton Regional	2014	C
58	7.411	13.88	0.89	2	0.00	2806.15	290	North Burnett Regional	2017	C
59	0.000	13.87	0.90	2	0.00	2786.93	146	Brisbane City	2014	H
60	6.619	13.83	0.90	2	0.00	2740.02	297	Ipswich City	2016	C
61	0.456	13.82	0.90	2	0.00	2730.82	298	Toowoomba Regional	2016	C
62	0.981	13.71	0.92	2	0.00	2583.44	315	Townsville City	2008	C
63	1.552	13.50	0.94	2	0.00	2323.39	178	Tablelands Regional	2016	C
64	6.931	13.23	0.97	2	0.00	2026.45	211	Cassowary Coast Regional	2007	C
65	0.399	13.07	0.99	2	0.00	1875.36	228	Western Downs Regional	2006	C
66	6.734	13.01	0.99	2	0.00	1813.87	228	Scenic Rim Regional	2016	C
67	8.480	12.93	0.99	2	0.00	1742.58	467	Western Downs Regional	2007	C
68	0.000	12.71	0.99	2	0.00	1564.97	260	Isaac Regional	2017	H
69	0.880	12.65	1.00	2	0.00	1520.45	272	Whitsunday Regional	2014	C
70	0.475	12.55	1.00	2	0.00	1440.98	287	Ipswich City	2014	C
71	6.271	12.51	1.00	2	0.00	1415.28	575	Scenic Rim Regional	2015	C
72	0.237	12.41	1.00	2	0.00	1342.74	308	Ipswich City	2002	C

ID	Radius (Km)	LLR	P value	Observed cases	Expected cases	RR	Population	LGA	YEAR	Cluster

LLR: log-likelihood ratio; RR: relative risk; LGA: Local Government Area
